# Targeting the NLRP3 Inflammasome via BTK

**DOI:** 10.3389/fcell.2021.630479

**Published:** 2021-02-25

**Authors:** Alexander N. R. Weber

**Affiliations:** ^1^Interfaculty Institute for Cell Biology, Department of Immunology, University of Tübingen, Tübingen, Germany; ^2^iFIT – Cluster of Excellence (EXC 2180) “Image-Guided and Functionally Instructed Tumor Therapies”, University of Tübingen, Tübingen, Germany; ^3^CMFI – Cluster of Excellence (EXC 2124) “Controlling Microbes to Fight Infection”, University of Tübingen, Tübingen, Germany; ^4^Deutsches Konsortium für Translationale Krebsforschung (DKTK; German Cancer Consortium), Partner Site Tübingen, Department of Immunology, University of Tübingen, Tübingen, Germany

**Keywords:** Bruton's tyrosine kinase, NLRP3 inflammasome, inflammation, phosphorylation, Interleukin-1 (IL-1), kinase inhibitor

## Abstract

The NLRP3 inflammasome represents a critical inflammatory machinery driving pathology in many acute (e. g., myocardial infarction or stroke) and chronic (Alzheimer's disease, atherosclerosis) human disorders linked to the activity of IL-1 cytokines. Although the therapeutic potential of NLRP3 is undisputed, currently no clinically approved therapies exist to target the NLRP3 inflammasome directly. The recent discovery of BTK as a direct and positive regulator of the NLRP3 inflammasome has, however, raised the intriguing possibility of targeting the NLRP3 inflammasome via existing or future BTK inhibitors. Here, I review the mechanistic basis for this notion and discuss the molecular and cellular role of BTK in the inflammasome process. Specific attention will be given to cell-type dependent characteristics and differences that may be relevant for targeting approaches. Furthermore, I review recent (pre-)clinical evidence for effects of BTK inhibitors on NLRP3 activity and highlight and discuss open questions and future research directions. Collectively, the concept of targeting BTK to target NLRP3-dependent inflammation will be explored comprehensively at the molecular, cellular and therapeutic levels.

## Introduction

**Figure 1:**
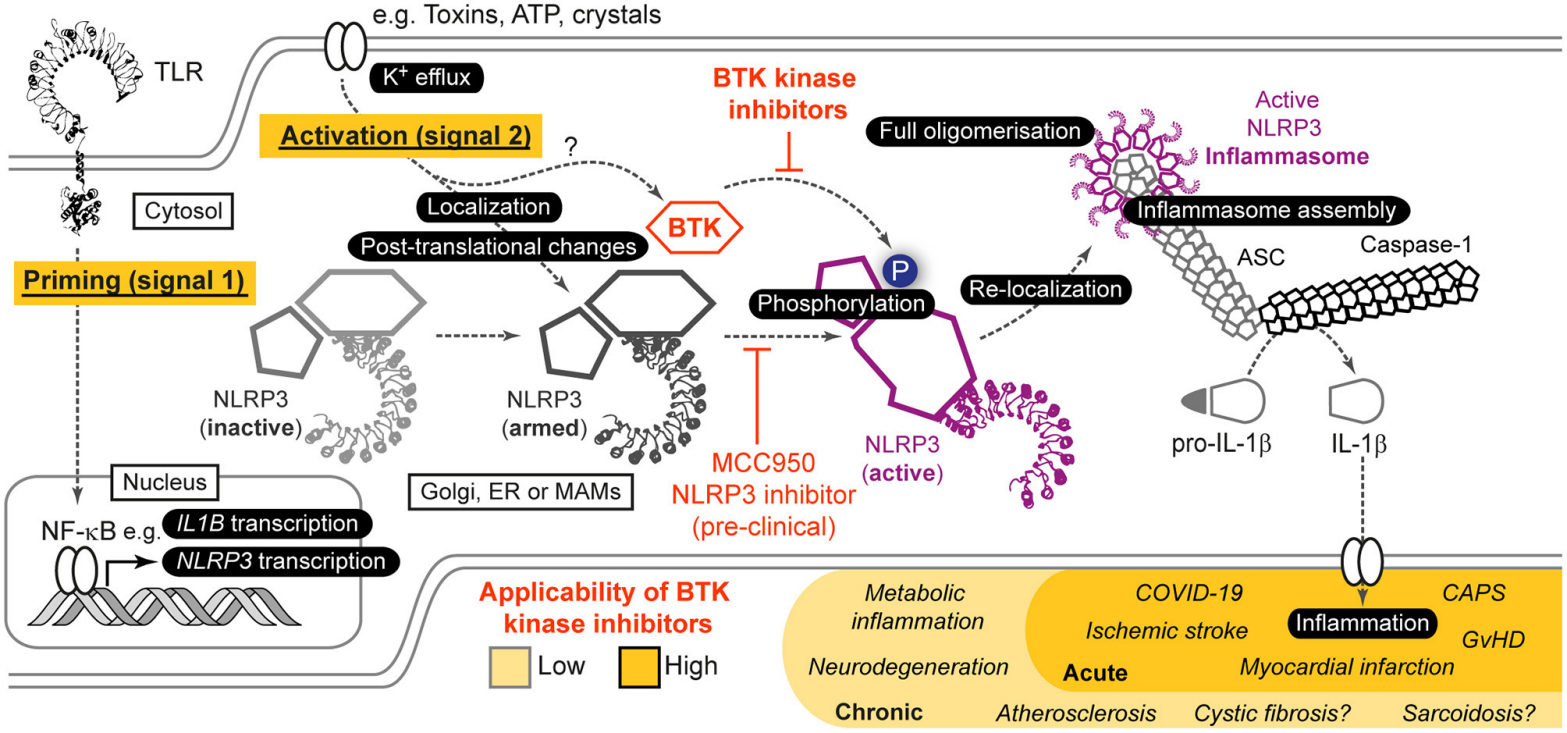
Role of BTK in NLRP3 inflammasome activation and resulting opportunities for indirect targeting of NLRP3 via BTK. Simplified scheme of NLRP3 activation highlighting the role of BTK. For further details see main article and Weber et al. (2020). Based on *in vitro*, pre-clinical mouse models as well as *ex vivo* analyses in human samples and patients, indirect targeting of NLRP3 via current BTK inhibitors could be envisaged especially in acute NLRP3/IL-1 axis-mediated inflammatory conditions, whereas in chronic conditions NLRP3-selective targeting would be necessary.

Within the immune system, Bruton's tyrosine kinase (BTK) appears to be something like a “Swiss Army knife,” a highly versatile molecule that seemingly participates in innumerable processes [reviewed in Weber et al. (2017)]: These range from immune cell development and differentiation in neutrophils and B cells (Khan et al., 1995; Fiedler et al., 2011), to innate functions e.g. Toll-like receptor (TLR), Fc, and growth factor receptor signaling, phagocytosis, and platelet activation (Quek et al., 1998; Horwood et al., 2006; Jongstra-Bilen et al., 2008; Melcher et al., 2008; Singhal et al., 2011; Strijbis et al., 2013), and to adaptive immunity, e.g. BCR signaling (Wilson et al., 2015). To add to this, BTK is not only relevant in normal cells but also in the context of malignancy, most notably as a target for B cell malignancies (Wilson et al., 2015). Recent evidence regarding novel splice variants of BTK in colon (Grassilli et al., 2016) and breast (Eifert et al., 2013) cancer expand its significance from the well-known and critical role of BTK in malignant B cells. It is the latter that has driven intense research and clinical development of pharmacological inhibitors to target the kinase activity of BTK. Ibrutinib was the first inhibitor approved by the FDA for the treatment of chronic lymphocytic leukemia and mantle cell lymphoma (Byrd et al., 2013; Mcnally et al., 2015) and later steroid-resistant chronic Graft-vs.-Host disease (GvHD) (Jaglowski and Blazar, 2018), but multiple other molecules are now in development or clinical testing. So the coming years are not only going to witness better targeting of B cell malignancies but a host of new applications especially in innate immunity await exploration. Especially in innate immunity, one process has recently gained considerable interest as a regulator of inflammation, the NLRP3 inflammasome (Agostini et al., 2004; Swanson et al., 2019; Weber et al., 2020). NLRP3 is a cytoplasmic danger and stress sensor belonging to the Nod-like receptor family of pattern recognition receptors (PRRs) (Agostini et al., 2004; Takeuchi and Akira, 2010), that is activated by homeostasis-perturbing exogenous and endogenous cues. Its expression has been reporter for multiple myeloid cells, ranging from macrophages (Franchi and Nunez, 2008), dendritic cells (Ghiringhelli et al., 2009), platelets (Murthy et al., 2017) and neutrophils (Mankan et al., 2012) to microglia (Freeman et al., 2017), Kupffer cells (Huang et al., 2013) and cardiomyocytes (Yao et al., 2018) [summarized in Guarda et al. (2011) and Weber et al. (2020)]. At least *in vitro*, NLRP3 activation involves a transcriptional and post-translational priming phase (signal 1), e.g., via TLR signaling, followed by an actual activation step (signal 2), which has been linked to K^+^ efflux (Munoz-Planillo et al., 2013). The latter leads to conformational changes in NLRP3 (Tapia-Abellan et al., 2019) and the assembly of a large multi-protein complex termed the inflammasome (Figure 1) (Agostini et al., 2004; Swanson et al., 2019). The NLRP3 inflammasome additionally includes an adaptor, ASC, and the enzyme pro-caspase-1 (Swanson et al., 2019). Upon assembly, caspase-1 becomes active and cleaves inactive IL-1 cytokine family members to mature into biologically active and secreted forms that potently trigger inflammation (Swanson et al., 2019). Comprehensive reviews of NLRP3 activation and regulation are found here (Mangan et al., 2018; Swanson et al., 2019; Weber et al., 2020). Inflammation instigated by NLRP3 has been shown to be fundamental to pathophysiological changes in diseases like cryopyrin-associated periodic fever syndrome (CAPS) (Agostini et al., 2004), myocardial infarction (Abbate et al., 2015), stroke (Ito et al., 2015), liver inflammation (Wree et al., 2014), type 2 diabetes (Masters et al., 2010), Alzheimer's disease (Heneka et al., 2013) Parkinson's disease (Gordon et al., 2018) and aging (Swanson et al., 2019; He et al., 2020; Weber et al., 2020). Hence, targeting NLRP3 is one of the most prominent therapeutic goals in innate immunity (Mangan et al., 2018). Unfortunately, there are so far no clinically approved inhibitors for direct targeting of NLRP3 or caspase-1 (Mangan et al., 2018; Swanson et al., 2019). When others and us recently discovered BTK as a novel positive regulator (Ito et al., 2015; Liu et al., 2017) this posed the intriguing possibility to target NLRP3-mediated inflammation using BTK kinase inhibitors (Banoth and Cassel, 2017; Henrickson, 2017; Liu et al., 2017). I here review the mechanistic and clinical basis for this notion, current controversies and open questions.

## Molecular Contributions of BTK to the NLRP3 Activation Process

BTK was identified as a NLRP3 regulator by two independent groups based on tyrosine kinase inhibitor and phospho-proteomics screens (Ito et al., 2015; Liu et al., 2017): Ito et al. (2015) showed a positive regulatory role for BTK in murine myeloid cells and its targeting reduced NLRP3 inflammasome-dependent IL-1β release. Similar results were obtained in our lab for primary human immune cells (Liu et al., 2017). Both studies also showed a physical interaction of BTK with NLRP3 and its adaptor ASC in overexpression systems (Ito et al., 2015; Liu et al., 2017), hinting to a direct role in the inflammasome process. BTK seemed specific for NLRP3 as other inflammasomes were not affected (Ito et al., 2015). Interestingly, BTK was rapidly phosphorylated upon NLRP3 activation, suggesting that its kinase activity might be relevant for the NLRP3 activation process. We therefore investigated whether BTK also interacted with endogenous NLRP3 in primary immune cells. Indeed, an interaction was detected after LPS stimulation of cells, i.e., after initiation of the priming phase (signal 1) and before the actual activation step (signal 2). Furthermore, we noted that NLRP3 became phospho-tyrosine-modified upon activation of NLRP3 by nigericin, in our hands (Bittner et al., 2020, Preprint) and also observed in Mao et al. (2020). This modification was BTK-dependent as it was reduced in BTK-ablated murine *Btk* KO BMDM and human XLA PBMC or inhibitor treated cells (Bittner et al., 2020, PrePrint). We subsequently mapped BTK-modified tyrosine residues and found that BTK was able to modify at least four tyrosine residues—three located in a critical localization motif of NLRP3, the so-called polybasic motif (PBM), and one adjacent to this motif (Bittner et al., 2020, PrePrint). The positively charged PBM was shown to direct NLRP3 toward phospho-inositol-4-phosphate (PIP4)-rich membranes (e.g., Golgi and possibly endosomes or mitochondria-associated membranes) via charge interactions (Zhang et al., 2017; Chen and Chen, 2018; Seoane et al., 2020). On these membranes, NLRP3 is thought to oligomerize (Chen and Chen, 2018) but then dissociate and re-locate to the microtubule-organizing center (MTOC) where ASC and NEK7 are engaged (Magupalli et al., 2020). On a peptide level, phospho-modification of tyrosine residues in the PBM altered the charge of this region (Bittner et al., 2020, PrePrint) and therefore may support the translocation or release of NLRP3 from PI4P-rich membranes, which would enable HDAC-6/dynein-dependent transport toward the MTOC where NLPR3 was shown to assemble a macromolecular ASC- and caspase-1 containing inflammasome (Magupalli et al., 2020). This would be similar to the described function of Protein Kinase D to promote membrane dissociation (Zhang et al., 2017), albeit the latter mechanism appears PBM-independent as the modified site, S295, locates outside the PBM, but may also involve charge repulsion upon serine phosphorylation. In line with this notion of BTK-mediated phosphorylation to alter PBM charge, mutation of the BTK-modified tyrosines in overexpressed NLRP3 resulted in lower binding to PIP4 beads (Bittner et al., 2020, Preprint). Furthermore, BTK inhibition or genetic ablation in primary cells coincided with a reduced ability of NLRP3 to form oligomers and to engage ASC (Bittner et al., 2020, Preprint). Furthermore, we observed that a mutant form of NLRP3, in which the modified tyrosines were mutated to phenylalanine failed to induce IL-1 release (Bittner et al., 2020, Preprint), indicating that these tyrosine positions indeed are vital for full NLRP3 activation. Collectively, studies by us (Liu et al., 2017; Bittner et al., 2020, Preprint) and others (Ito et al., 2015) suggest that BTK is a direct and positive regulator of the inflammasome process, raising the intriguing notion of targeting NLRP3-mediated inflammation directly via BTK (Banoth and Cassel, 2017; Henrickson, 2017; Liu et al., 2017).

## Targeting NLRP3 Indirectly Via BTK—a Viable Therapeutic Opportunity in the Absence of Clinically Approved Direct NLRP3 Inhibitors

Given its involvement in multiple inflammatory disorders, multiple strategies for targeting NLRP3-mediated inflammation have been proposed. FDA-approved since 2001 is the targeting of IL-1 itself via either recombinant IL-1 receptor antagonist (IL-1RA, tradename Rilonacept) or monoclonal antibodies (tradenames Anakinra and Canakinumab) (Dinarello and Van Der Meer, 2013). These treatments are well-established and show good efficacy; however, they involve regular injections, suffer from resistance mechanisms such as anti-drug antibodies, and do not target NLRP3 directly; rather, they only target one (IL-1) of the several NLRP3-regulated inflammatory alarmins (e.g., IL-18 and HMGB-1) (Wiken et al., 2018) and do not affect pyroptosis. The development of direct NLRP3 inhibitors hence is receiving much attention and industrial efforts, especially since the discovery of MCC950 (also known as CRID3) as a direct NLRP3 inhibitor (Coll et al., 2015). MCC950 binds to the NACHT domain and stabilizes the closed, i.e., inactive, conformation of NLRP3, thus blocking its activity (Coll et al., 2019; Tapia-Abellan et al., 2019). A growing number of pre-clinical *in vivo* models have since reported efficacy for MCC950, e.g., in models of cryopyrin-associated periodic syndrome [CAPS (Coll et al., 2015)], Alzheimer's disease [APP/PS1 model (Dempsey et al., 2017)], sarcoidosis [trehalose 6,6'-dimycolate-granuloma model (Huppertz et al., 2020)], atherosclerosis [ApoE model (Van Der Heijden et al., 2017)] or cystic fibrosis [*Cftr* transgenic model (Mcelvaney et al., 2019)].

However, despite several clinical studies, so far no direct NLRP3 inhibitor resembling MCC950 has reached late stage clinical development or approval. Thus, alternative strategies will be of high interest for probably at least the next 5–8 years. Targeting NLRP3 indirectly via BTK could be one of them. It would build upon the fact that BTK is a well-established apharmacological target and that pharmacological inhibitors are either already FDA-approved (ibrutinib, acalabrutinib, and zanubrutinib) or in late-stage clinical development. Compared to anti-IL-1 therapy, inhibiting BTK probably would still target NLRP3 proximally enough to block all known NLRP3-dependent effects, both in terms of alarmins and pyroptosis. Of course similar indirect NLRP3 targeting approaches could be envisaged for other known direct NLRP3 regulators [e.g., protein kinase D (Zhang et al., 2017)], albeit none of the known ones are clinically revant to date.

## Evidence From Preclinical *IN VIVO* Models for the Plausibility of BTK-Focussed NLRP3 Targeting

Insights into the plausibility of the targeting NLRP3 via BTK firstly came from our description of a reduced NLRP3 activity in patients on ibrutinib therapy (Liu et al., 2017). In these patients, who received a daily oral dose of ibrutinib of 420–560 mg, nigericin- and ATP-dependent IL-1 cleavage and release were reduced, whereas TNF release was comparable (Liu et al., 2017). We also observed a moderate effect in an *S. aureus in vivo* infection model, where ibrutinib administration led to reduced bacterial control (Liu et al., 2017). More prominent are the data by Ito et al. (2015) in an experimental model of ischemic stroke. They reported that ibrutinib suppressed infarct volume growth and neurological damage in line with reduced maturation of IL-1β and caspase-1 activation in infiltrating macrophages and neutrophils in the infarcted area. More recently, cardiac failure in the wake of sepsis was studied in an experimental model under ibrutinib and acalabrutinib administration (O'Riordan et al., 2019). *Btk*-deficient *Xid* mice were also protected in a polymicrobial sepsis model, with reduced NLRP3 activation contributing to the phenotype (O'Riordan et al., 2020). Interestingly, BTK inhibition reduced NLRP3 protein levels and IL-1 in the serum. Moreover, Purvis et al. (2020) described that ibrutinib treatment ameliorated the NLRP3 contribution to inflammation upon high-fat-diet in mice, which resulted in improved glycemic control. In stark contrast is a recent study proposing BTK as a negative regulator, based on increased intestinal inflammation in a BTK-inhibited or-deficient context (Mao et al., 2020): In this study, high LPS concentrations used for priming led to increased IL-1 release under conditions of BTK inhibition and in a colitis model, BTK deficiency was associated with greater inflammation. However, the ablation of regulatory B cells by BTK-deficiency (Yanaba et al., 2011; Kondo et al., 2018) or the intestinal involvement of other inflammasomes (e.g., NLRC4, NLRP6, pyrin or AIM2) (Elinav et al., 2011; Romberg et al., 2014; Hu et al., 2015; Sharma et al., 2018), which are not BTK-dependent (Ito et al., 2015), both could explain why IL-1 inhibition *in vivo* could counteract the seemingly negative regulatory role of BTK proposed (Mao et al., 2020). In humans, effective suppression of the NLRP3/IL-1 axis by BTK inhibitors, and thus a positive regulatory effect of BTK on NLRP3, was instead suggested by a recent off-label trial of acalabrutinib in COVID-19 patients (Roschewski et al., 2020). The improvement of patients under ventilation or oxygenation treatment was attributable to BTK function in macrophages, but not B cells (Roschewski et al., 2020), implicating BTK function in these cells and hence the NLRP3 inflammasome. Although in most of these studies, mechanistic details, e.g., phosphorylation of NLRP3 or interaction or phosphorylation of BTK, need to be clarified, a case for intervening in NLRP3-mediated inflammation at the level of BTK can clearly be made (Figure 1).

## Discussion

Given the effect of BTK inhibition on B cell development and function, the applicability of targeting NLRP3 via current BTK inhibitors at this stage appears limited to and most suitable for acute settings like myocardial infarction, stroke or COVID-19 rather than chronic NLRP3-mediated diseases such as Alzheimer's or Parkinson's disease. If strategies could be found that can discriminate between BTK function in B cells and macrophages, e.g., by targeted delivery or inhibitors based on protein-protein interactions, more long-term conditions may also come into view therapeutically. On the back of the promising studies described above, further mechanistic work seems mandatory. Additionally, in pre-clinical *in vivo* models conditional BTK alleles that circumvent the simultaneous ablation of B cell function characteristic for BTK KO mice will be highly desirable. These should be complemented by *ex vivo* studies of NLRP3 activity in cancer patients treated with the increasingly specific BTK inhibitors or biomaterial from healthy volunteers and XLA patients. Furthermore, one needs to bear in mind that BTK is only a partial NLRP3 regulator and that some IL-1 can still be released in the absence of BTK, albeit at drastically lower levels (Liu et al., 2017). Evidence from ibrutinib-treated patients (Liu et al., 2017) indicates that sufficient NLRP3 blockade may be clinically achievable via BTK inhibitors at tolerable doses, but prospective off-label clinical studies would be mandatory for the inflammatory setting of interest. Although BTK-NLRP3 interactions have been studied mainly in macrophages, effects of BTK inhibitors on NLRP3 function in platelets, neutrophils, Kupffer cells or microglia will be important to study for capturing the effect of systemic application of BTK inhibitors to block NLRP3-mediated inflammation in the absence of clinically approved NLRP3 inhibitors. Collectively, targeting the NLRP3 inflammasome via BTK represents an attractive yet fully-to-be-explored alternative application of BTK inhibitors to pathological inflammatory states beyond B cell malignancies. In fact, the clinical efficacy of BTK inhibitors in GvHD (Jaglowski and Blazar, 2018) and COVID-19, and the reported role of the NLRP3/IL-1 axis in these disease states (Jankovic et al., 2013; Roschewski et al., 2020) lend support to the notion that BTK-inhibitor therapy for NLRP3 inhibition warrants further preclinical and clinical exploration.

## Author Contributions

The author confirms being the sole contributor of this work and has approved it for publication.

## Conflict of Interest

The author declares that his laboratory received funding from Novartis during the course of the generation of this review. The funder was not involved in the collection, analysis, interpretation of data, the writing of this article or the decision to submit it for publication.
